# Publication trends in journal of clinical and experimental dentistry

**DOI:** 10.4317/jced.56640

**Published:** 2020-09-01

**Authors:** Vidhi-Kiran Bhalla, Sherin-Jose Chockattu

**Affiliations:** 1 Senior Lecturer, Department of Conservative Dentistry and Endodontics ITS centre for dental studies and Reasearch, Ghaziabad, Uttar Pradesh; 2 Senior Lecturer ,Department of Conservative and Endodontics , Bapuji Dental College and Hospital, Davangere, Karnataka

## Abstract

**Background:**

Journal of Clinical and Experimental Dentistry (J Clin Exp Dent; JCED) is an English language journal published by the Spanish Society of Oral Surgery, and has been online since 2009. It is indexed in PubMed Central and Scopus since 2012, with monthly publications since 2016. The purpose of this article was to review and analyse the publications in this journal since its inception, over a period of 11 years (2009-2019).

**Material and Methods:**

This paper assessed the number, type and subjects of the articles published in the journal over 11 years. The institutions of the first authors, number of PubMed citations and the Hirsch (h5) index was assessed and analysed.

**Results:**

The manuscripts published in JCED have gradually increased over the years, with Original research articles accounting for the bulk of contributions. The journal publishes articles mainly from the subjects of Oral Pathology and Operative Dentistry and Endodontics. Articles published in JCED are indexed in PubMed Central (since 2012), Scopus, DOI system, and Google Scholar. A country-wise mapping of the (first) author’s institutions revealed significant contributions from researchers from all over the world. With an h5 index of 26, the journal was ranked among the top six multispeciality journals. The most cited articles were the literature reviews on common oral lesions (recurrent apthous stomatitis and candidiasis).

**Conclusions:**

The journal has contributed to the growth of scientific literature pertaining to subjects from all the fields of dentistry. Over the past 11 years, JCED has served as a platform for large number of manuscripts in all the disciples of dentistry, from researches all over the world.

** Key words:**Publication trends, Journal of Clinical and Experimental Dentistry, Bibliometrics.

## Introduction

Journal of Clinical and Experimental Dentistry (J Clin Exp Dent) is the official journal of the Spanish Society of Oral Surgery (SECIB) with the ISSN 1989-5844.This journal has been online since September 2009, under the leadership and guidance of Prof. José V. Bagán, the Editor-in-Chief (1). In the year 2010, the issues were published quarterly, and since 2011, the journal initiated bimonthly publications until 2016. From 2017 onwards, issues are put forth every month, and it continues this trend till date. This can be attributed to the spurt in manuscript submissions over the years. The journal is indexed in Pubmed and Pubmed Central (since 2012), Scopus, DOI system, and Google Scholar (1).

J Clin Exp Dent is a peer-reviewed, open access journal providing recent and innovative research and development pertaining to all the nine specialities of dentistry, which includes Periodontology, Community and Preventive Dentistry, Esthetic Dentistry, Biomaterials and Bioengineering in Dentistry, Operative Dentistry and Endodontics, Prosthetic Dentistry, Orthodontics, Oral Medicine and Pathology, Odontostomatology for the disabled or special patients, and Oral Surgery. Pertaining to the types of articles, the journal publishes original research, case reports and review articles (1).

The application of bibliometric data is useful measure to assess the performance of authors, scientific articles, journals or institutions (2,3). Citation-based measures that enable this assessment include Impact Factor (IF; Clarivate Analytics’ Journal Impact Factor) and the Hirsch index (h5-index). Impact factor is a reflection of the number of citations of the journal’s articles during the preceeding two years. The h5-index attempts to measure the impact and productivity of the published work of the journal/ researcher over a period of five years (4).

An assessment is yet to be conducted on the nature of articles published in JCED and their citations, nor has been the journal been compared with other speciality journals. An analysis and study of publication patterns and citations of a journal is necessary for evaluation of the journal’s contribution to the growth of scientific literature. Hence, the aims of the present article were: 1) to perform an assessment of the articles published from 2009-2019 based on their type, subjects and authorship; and 2) to analyse the citation of JCED journal articles and compare it with other speciality journals.

## Material and Methods

From the year 2009 to 2019, using the official website of JCED (http://www.medicinaoral.com/odo/indice.htm) (5), the eleven volumes and fifty eight issues were analysed for number, type, subject of articles, the country of the first author, and the article citations (since 2012) in PubMed.

On the Google Scholar website, under “metrics”, (https://scholar.google.com/citations?view_op=top_venues&hl=en) (6) a search for “Journal of Clinical and Experimental Dentistry” was done to determine the h5 index or Hirsch index of the journal. Using the SCimago Journal & Country Rankings website (https://www.scimagojr.com/journalrank.php?category=3501), (7) a search was made for the multispeciality journals and journals in the speciality of Restorative Dentistry/ Endodontics/ Oral Pathology, and their h5 indices were compared with JCED in Google Scholar. Considering the scope of the journal, all the relevant journals were selected and compared.

The aforementioned analysis was carried out by both the authors independently, with discrepancies being resolved by consensus.

## Results

From 2009-2019, 10 volumes and 80 issues of the JCED journal were analysed. Initially, the number of articles published was highly variable; subsequently there was a consistent increase in the number over the years. The maximum number of articles (205) were published in 2017, pertaining to all the fields of dentistry, with maximum contribution from the speciality of Oral pathology (32.3%) and Operative Dentistry and Endodontics (17.8%). This was followed by articles from the fields of Oral surgery (11.4%), Orthodontics (8.8%), Prosthodontics (7.5%), Community & Preventive Dentistry (7.4%), Periodontology (4.4%) and Biomaterials and Bioengineeering (4.4%). The journal received a minor contribution from the fields of Esthetic Dentistry (3.2%), Odontostomatology for the disabled/ special patients (1.3%), as well as Oral Medicine and Radiology (0.5%) (Table 1).

**Table 1:**
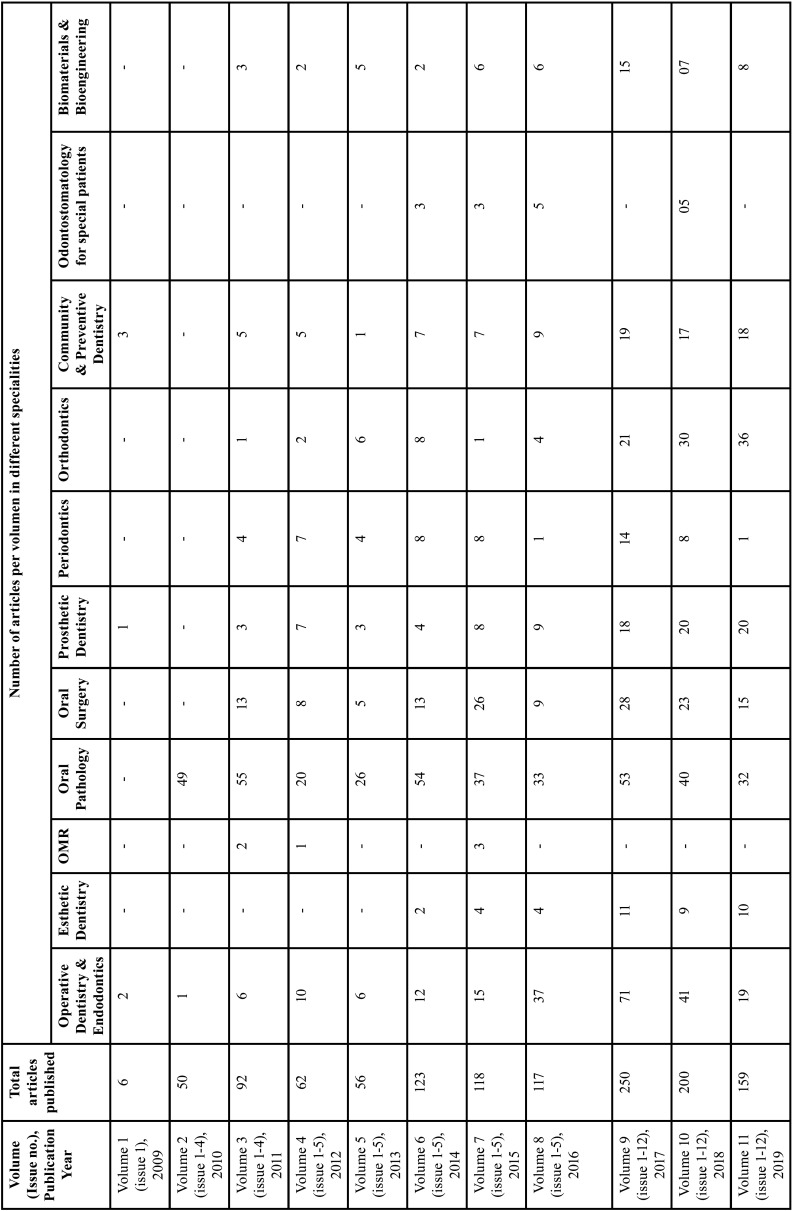
Articles published in JCED in different specialities.

An analysis of the type of articles published revealed an overwhelming majority of Original research articles (66.8%), followed by Case reports (20%) and Reviews (13.2%) (Table 2). A total of 30 systematic reviews have been published over the past 10 years.

**Table 2:**
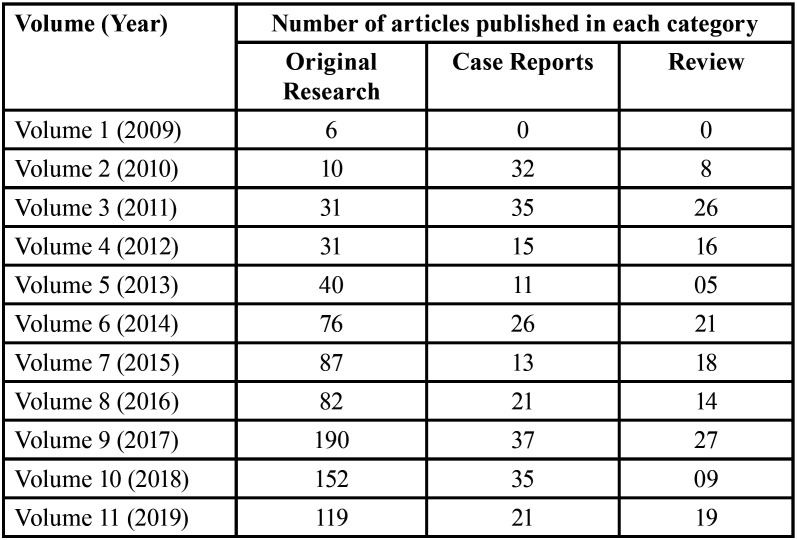
Number of articles published in each category in JCED (2009-2019).

**Figure 1:**
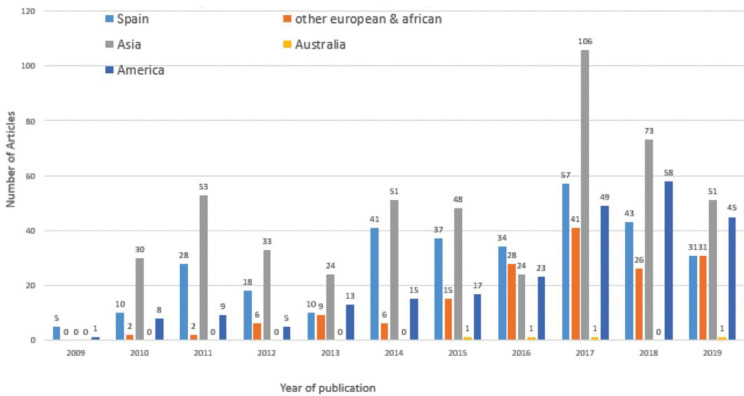
Institutions of the first author by country.

A country-wise assessment was performed on the corresponding author’s institution. Unlike in 2009, the year of inception of the journal where the vast majority of articles were from Spain, the succeeding years witnessed increasing contributions from other countries. By 2017, Asian nations (Iran, Turkey, Malaysia, India, Thailand, Japan and Saudi Arabia) accounted for the majority of the articles published, with increasing contributions from Europe (Italy, Portugal, Greece, France), Africa (Egypt, Nigeria, Libya) and the Americas (USA, Canada, Brazil). However, the contributions from Spain (the native country) still represented the majority (Fig. 1).

**Figure 2:**
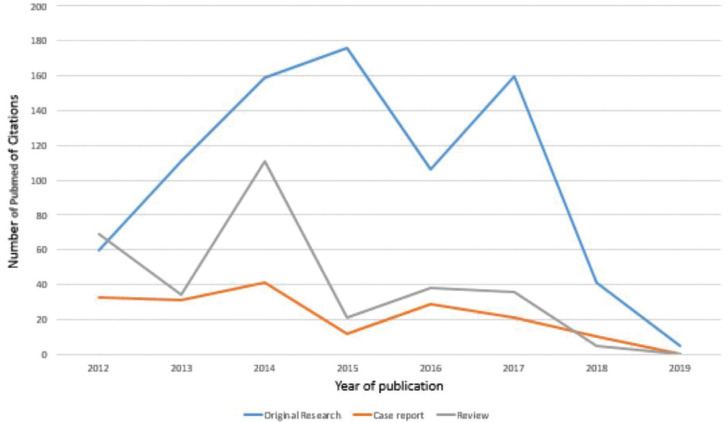
Number of Pubmed Citations in JCED in each category (2012-2019).

**Figure 3:**
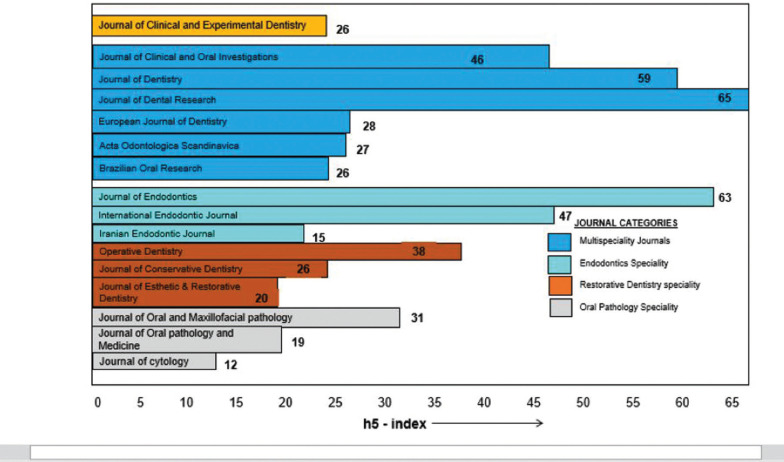
Comparison oj JCED with other journals in the speciality.

Articles published in JCED have been cited in PubMed (since 2012). The number of citations showed a sharp spike in 2014 with over 304 citations, including Original research, reviews and case reports. In general, original research articles have been cited more consistently than case reports and reviews (Fig. 2).The number of citations were analysed for each article, using the PubMed Website. The 10 most cited articles of JCED in PubMed and Google Scholar (as on Nov 2019) is given in Table 3, Fig. 3. The most cited articles have been literature and systematic reviews on the recent treatment aspects of oral lesions like candidiasis and recurrent apthous ulcers.

**Table 3:**
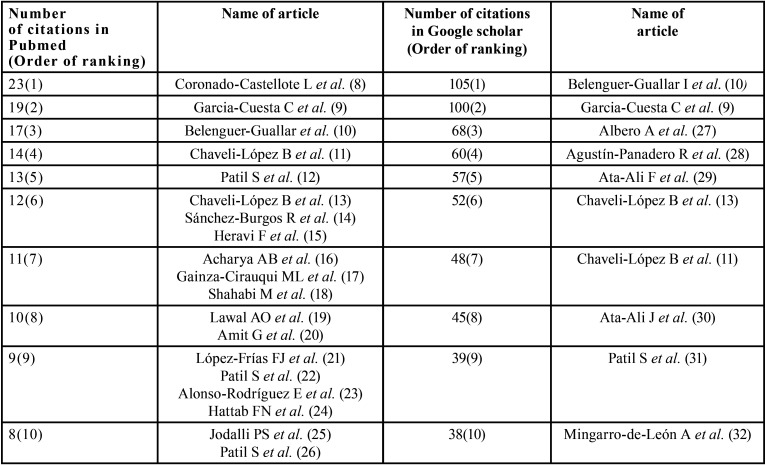
Number of citations of JCED in Pubmed and Google Scholar.

On comparing the h5 index of the journal to other multispeciality journals, endodontic and oral pathology journals, JCED ranks amongst the top six, with an h5 index of 26. Also, the h5 index was similar to Brazilian Oral Research journal and Journal of Conservative Dentistry.

## Discussion

In our assessment of publication trends of JCED, over a period of 11 years (2009-2019), an important factor which was noted was the large number of quality peer reviewed original research articles, case reports and reviews. The manuscripts published span the whole spectrum of dentistry covering all the specialities and a wide range of topics. Although, the initial issues had maximum contribution from the speciality of Oral Medicine and pathology, the subsequent issues included a more uniform distribution of subjects, including a separate section for the diagnosis and management dental issue concerning of disabled or special patients.

JCED has witnessed a significant growth in scientific literature in the recent years. Whilst the bulk of articles have been from Spain, there has been a spurt in contribution from researchers of the other European nations, along with Asian and American nations. The trend of globalisation has been a significant factor in the journals success (33). The indexing of a journal in relevant and prominent databases provides visibility and allows dissemination of information published through such journals to researchers in the field (34) Currently, JCED is indexed/ abstracted in PubMed and PubMed Central (since 2012), Scopus, DOI system, and Google Scholar (1).

The journal has publications from all the sub-specialities of dentistry, the major contribution from the field of Oral Pathology, and Operative Dentistry and Endodontics. An analysis of the individual contributions of the specialities to the journal’s scientific progress was not done. Additionally, we have taken into account only the institution (or country) of the corresponding author, although in many articles, there have been contributions from authors of different countries.

The Impact factor (IF) published by the Thomson Reuters’ annual Journal Citation Reports (JCR) is a popular and frequently used bibliometric index (35). It is fundamentally the average number of citations per year published over the previous 2 years, thereby measuring the journal’s contribution and scope to the research field. However, the extent to which IF is appropriate for the evaluation of the quality of a specific article or journal and for the evaluation of individual or collective research achievements is highly debaTable and often considered biased (36,37).

The Hirsch index (h-index) is a measure of author’s (or journal) output in terms of quality and consistency, based on the total number of publications and the total number of citations received of that work. It is traditionally measured over a five year period, hence it is referred to as “h5 index” (4). A comparison of the h5 index of JCED with other multispeciality journals revealed that JCED ranks amongst the top six multispeciality journals.

The number of citations in Pubmed showed a spike in 2014 with an overall 167 citations in original research, 96 citations in Reviews, and 41 in case reports. The general trend was more number of citations for research articles. Citations decreased thereafter, which was again followed by a slight increase in 2017.

An analysis of the most cited articles was done in PubMed and Google Scholar (Table 3). The most cited article on Google Scholar has been the literature review on the treatment of recurrent aphthous stomatitis; while on PubMed it has been the review on clinical and microbiological diagnosis of candidiasis. The information gathered in these reviews play a crucial role in updating the knowledge on the most common oral mucosal lesions encountered in clinical practice, relevant properties of dental materials, dental management of medically-complex patients, and forensic dentistry. Amongst the original research articles, prevalence of dental anomalies and retrospective studies on keratocystic odontogenic tumours have been the most cited. The journal has published numerous rare and interesting case reports on odontogenic tumours, oro-facial syndromes and unusual developmental disturbances. The most cited case report has been on schwannoma of the hard palate.

## Conclusions

Over the last 11 years, since its genesis, JCED has served as a platform for a large number of manuscripts, in all the nine specialities of dentistry from researchers from all over the world. The unrestricted access and the nominal fee charged for publication has encouraged researchers and students from all over the world to submit their research papers and review articles to the journal.
